# Rs2200733 and rs10033464 on chromosome 4q25 confer risk of cardioembolic stroke: an updated meta-analysis

**DOI:** 10.1007/s11033-013-2707-z

**Published:** 2013-09-25

**Authors:** Yan-yan Cao, Fei Ma, Yan Wang, Dao Wen Wang, Hu Ding

**Affiliations:** 1 Institute of Hypertension and Department of Internal Medicine, Tongji Hospital, Tongji Medical College, Huazhong University of Science and Technology, 1095# Jiefang Ave, Wuhan, 430030 People’s Republic of China; 2 Genetic Diagnosis Center, Tongji Hospital, Tongji Medical College, Huazhong University of Science and Technology, Wuhan, 430030 People’s Republic of China

**Keywords:** Stroke, 4q25, Meta-analysis

## Abstract

A recent genome-wide association study elucidated that 4q25 was implicated in ischemic stroke, but subsequent studies showed inconsistent results. In order to get coincident conclusion, we investigated two SNPs (rs2200733, rs10033464) on chromosome 4q25 in 1,388 stroke patients and 1,629 controls from Chinese Han population and then performed a meta-analysis. Although we failed to detect any association between 4q25 and stroke in our case–control study, meta-analysis revealed that rs2200733 showed association with overall stroke (OR 1.18, 95 % CI 1.08–1.27), but not for rs10033464. Subsequently subgroup analysis indicated that both rs2200733 and rs10033464 conferred increased risk for cardioembolic stroke (CE stroke) (for rs2200733, OR 1.38, 95 % CI 1.26–1.51; for rs10033464, OR 1.14, 95 % CI 1.02–1.26), while rs2200733 was marginal associated with non-CE stroke (OR 1.09, 95 % CI 1.02–1.16). our results demonstrated that two SNPs (rs2200733 and rs1003346) on chromosome 4q25 were limited to the stroke of cardioembolic etiology. To confirm this conclusion, well-designed studies with larger sample size involving case–control populations with homogeneous ancestry warrant to be conducted in the future.

**Electronic supplementary material:**

The online version of this article (doi:10.1007/s11033-013-2707-z) contains supplementary material, which is available to authorized users.

## Introduction

Stroke is a multifactorial complex disorder causing long-term disability and increasing family responsibilities [1]. With high morbidity and mortality, stroke has become the first cause of death in China [2]. It was no doubt that environmental and clinical risk factors (such as hypertension, diabetes, hyperlipidemia, high body mass index (BMI) and smoking state etc.) contributed to the progress of stroke [3, 4], however twin and family history studies suggested that a genetic component also played an important role [5, 6]. Recently, a GWAS from Iceland found that rs2200733 and rs10033464 on chromosome 4q25 both were strongly associated with stroke [7]. However, a series of later studies failed to replicate this result and obtained inconsistent results [8–15]. Aiming to get reliable and stable conclusion about the relationship between 4q25 and stroke, we analyzed two SNPs (rs2200733 and rs10033464) on chromosome 4q25 in Chinese Han population, including 1,388 stroke patients and 1,629 controls. Then, we combined our study with previously published articles to conduct a meta-analysis to asses the relationship between these two SNPs and the risk of overall stroke and subtypes.

## Materials and methods

### Study subjects

This is a multicenter study sponsored by the Ministry of Science and Technology of China. Briefly, a total of 1,388 stroke patients were recruited in our study between November 2004 and January 2009 from five hospitals in Wuhan, China: atherothrombotic stroke (thrombosis, *n* = 716), lacunar infarction (lacuna, *n* = 407), and entracerebral hemorrhage (hemorrhage, *n* = 265). The inclusion criteria and description about stroke subjects have been previously reported [16]. Ethnically and geographically matched controls (*n* = 1,629) were randomly selected from the healthy, community-based residents by house-to-house recruitment. All the study protocols were approved by the review board of the Ministry of Public Health, Ministry of Science and Technology of China and the ethics committees at all participating hospitals (Tongji Hospital, Union Hospital, Xinhua Hospital, First Wuhan Hospital and Wugang Hospital), and informed written consent was obtained from all participants. Experiments were conducted according to the principles expressed in the Declaration of Helsinki.

### DNA isolation and genotyping

Genomic DNA was extracted using the QG-Mini80 workflow with a DB-S kit (FUJIFILM Corporation, Tokyo, Japan) as instructed. The SNPs were genotyped using the TaqMan SNP allelic discrimination on the TaqMan™ 7900HT Sequence Detection System under standard conditions as previously described [17]. Probe and primer sequences for these assays were custom designed by ABI Primer Expression 3.0 software and synthesized by GeneCore BioTechnologies Company, Limited, Shanghai, China (supplementary Table 1). Allelic discrimination was measured automatically using the Sequence Detection Systems 2.1 software (autocaller confidence level 95 %). A total of 10 % of all genotypes were repeated in independent PCRs to check for consistency and to ensure intraplate and interplate genotype quality control. No genotyping discrepancies were detected between the repeated samples. In addition, all the DNA samples for cases and controls were run in the same batch. In order to verify the accuracy of Taqman probe method for genotyping, we randomly selected 151 (about 5 % of sample size) samples from cases and controls to conduct direct DNA sequence analysis using the BigDye^®^ Terminator v3.1 Cycle Sequencing Kits on an ABI PRISM 3130xl Genetic Analyzer (Applied Biosystems, Foster City, CA, USA). At last, we found these two different genotyping methods were highly consistent.

### Statistical analysis

All quantitative variables were generally described as means with standard deviation (SD). one-way ANOVA test was performed to compare the baseline characteristics of different groups, such as age, BMI etc.; χ^2^ test was used for qualitative variables. Multiple unconditional logistic regression was used to estimate odds ratio (OR) and 95 % confidence interval (CI) under different genetic models after adjusting for gender, age, BMI, hypertension, diabetes, hyperlipidemia and smoking status. Haplotype frequencies for these two SNPs combinations were first estimated by *haplo.stats* (version 12.1) for the R statistical package and then verified using Haploview 4.0. Both of the software above uses the expectation–maximization (EM) algorithm when constructing the haplotypes. Global score tests were applied to evaluate overall haplotype frequency differences between cases and controls, whereas the haplotype-specific score tests were performed to test individual haplotype difference between cases and controls. To minimize the false-positive results generated from multiple statistical testing in our aforementioned analyses, we adopted the Bonferroni correction method for multiple testing.

### Literature search

We systematically searched in Pubmed, Embase and CNKI up until December, 2012 using the key words “stroke”, “ischemic stroke”, “cerebral infarction”, “cerebrovascular disease” paired with “4q25”, “SNP”, “PITX2”, “rs2200733” and “rs10033464”, respectively. Reference lists of relevant articles were also screened. The study inclusion criteria were as follows: (1) articles published in English or Chinese, (2) case–control or population-based studies, (3) studies with complete data on allele frequency and relevant clinical base characteristics, (4) studies about the association between rs2200733 or rs10033464 and stroke. Exclusion criteria were: (1) overlapping data, (2) case-only study, (3) small number cases, (4) review, (5) uncompleted article.

### Data extraction

Two investigators independently extracted useful data from the articles meeting the inclusion criteria, including first author name, publication year, number of stroke patients and controls, ORs (HRs) and 95 % CI. Disagreements were resolved by discussion.

### Statistical meta-analysis

All meta-analyses were performed by STATA software (version10.0). Pooled OR and 95 % CI were used to assess the association between rs2200733 or 10033464 and stroke. Heterogeneity was calculated by Cochran’s Q statistic and inconsistency index (I^2^). If probability values <0.10 or I^2^ > 0.50, heterogeneity was considered [18]; the random effects model was selected to estimate the pooled OR. Otherwise fixed effects model was applied. In order to reduce the heterogeneity of the meta-analysis, then, we performed subgroup analysis. Sensitivity analyses were used by omitting one cohort or one study at one time and calculating the pooled ORs of remaining cohorts or studies to assess the stability of our results. To test the publication bias, Begg’ funnel plot and Egger’ test were operated, All *P* values were two-tailed and *P* < 0.05 was considered statistically significant.

## Results

### Allelic association of SNPs in 4q25 and stroke

The characteristics of our study cohorts were shown in Table 1. Rs2200733 and rs10033464 genotypes were found to be in Hardy–Weinberg equilibrium in controls (both *P* > 0.05). Multivariate unconditional logistic regression analyses revealed that rs10033464 conferred risk for intracerebral hemorrhage in additive model (OR 1.29, 95 % CI 1.02–1.24, *P* = 0.038) and dominant model (OR 1.47, 95 % CI 1.08–1.97, *P* = 0.015); rs2200733 was not associated with overall stoke and subtypes (Table 2). However, none of associations remained significant with adjustment of Bonferroni correction.

**Table 1:** Baseline characteristics of stroke samples

Characteristics	Controls	Cases	Thrombosis	Lacunar	Hemorrage
	*n* = 1,629	*n* = 1,388	*n* = 716	*n* = 407	*n* = 265
Age (years)	59.29 ± 10.1	61.2 ± 10.4^a^	60.9 ± 10.4^a^	64.4 ± 9.0^a^	57.14 ± 11.0^a^
Male (%)	43.2	66.1^a^	66.9^a^	64.6^a^	66.6^a^
BMI (kg/m^2^)	24.2 ± 3.4	24.2 ± 3.3	24.3 ± 3.2	24.3 ± 3.5	23.8 ± 3.5
SBP (mmHg)	130.0 ± 20.7	148.3 ± 23.0^a^	147.6 ± 24.1^a^	145.4 ± 21.9^a^	154.5 ± 25.5^a^
DBP (mmHg)	80.3 ± 22.3	87.1 ± 13.8^a^	86.4 ± 14.6^a^	84.4 ± 12.6^a^	93.2 ± 16.7^a^
Hypertension (%)	28.1	68.7^a^	69.4^a^	68.8^a^	66.6^a^
Diabetes (%)	6.4	15.6^a^	18.9^a^	16.5^a^	5.3
Hyperlipidemia (%)	23.6	25.2	29.3	27.5	10.6^a^
Smokers (%)	27.1	51.1^a^	53.8^a^	50.6^a^	44.5^a^

Values are expressed as mean ± SD unless otherwise noted

*BMI* body mass index, *SBP* systolic blood pressure, *DBP* diastolic blood pressure

^a^Test for differences between cases and controls, *P* < 0.01

**Table 2:** Associations of SNPs with overall stroke and subtypes in different genetic models

SNPs	Subtypes	Minor allele frequency	ORs (95 % CI)	*P*	ORs (95 % CI)	*P*	ORs (95 % CI)	*P*
		Control/case	Additive model		Dominant model		Recessive model	
rs2200733	Thrombosis	0.473/0.467	1.03 (0.89–1.18)	0.705	1.00 (0.80–1.25)	0.998	1.08 (0.85–1.38)	0.518
	Lacuna	0.473/0.456	0.96 (0.81–1.14)	0.618	1.10 (0.84–1.44)	0.485	0.78 (0.57–1.05)	0.100
	Hemorrhage	0.473/0.458	0.98 (0.80–1.20)	0.817	1.08 (0.78–1.49)	0.636	0.85 (0.59–1.21)	0.358
	All	0.473/0.462	1.00 (0.89–1.13)	0.982	1.05 (0.87–1.26)	0.613	0.95 (0.78–1.13)	0.606
rs10033464	Thrombosis	0.209/0.207	1.05 (0.88–1.25)	0.612	1.09 (0.88–1.35)	0.412	0.89 (0.55–1.45)	0.636
	Lacuna	0.209/0.204	1.01 (0.82–1.25)	0.893	1.07 (0.83–1.38)	0.599	0.76 (0.40–1.43)	0.380
	Hemorrhage	0.209/0.236	1.29 (1.02–1.64)	0.038	1.47 (1.08–1.97)	0.015	1.11 (0.59–2.07)	0.748
	All	0.209/0.212	1.06 (0.92–1.22)	0.445	1.11 (0.93–1.32)	0.237	0.89 (0.60–1.32)	0.556

ORs and *P* value were estimated by multiple unconditional logistic regression after adjusting for gender, age, body mass index, hypertension, diabetes, hyperlipidemia and smoking status

### Haplotype analysis for these two SNPs

The correlation of two SNPs (rs2200733 and rs10033464) within the region of 4q25 is weak in our control data set (D′ = 0.91, r^2^ = 0.24). We used the *haplo.stats* program to determine whether the combined effects of two SNPs (rs2200733 and rs10033464) were associated with overall stroke and subtypes. As shown in Table 3, we failed to detect a significant difference for these two SNPs for all the subjects at haplotype levels, which was consistent with single SNP analysis.

**Table 3:** Association of haplotypes with overall stroke and subtypes

Haplotype^a^	Control	All stroke	*P* ^b^	Thrombosis	*P* ^b^	Lacunar	*P* ^b^	Hemorrhage	*P* ^b^
TG^c^	0.517	0.525	0.478	0.519	0.834	0.539	0.297	0.517	0.753
CG	0.275	0.265	0.36	0.275	0.927	0.275	0.388	0.248	0.175
CT	0.199	0.197	0.938	0.191	0.653	0.197	0.876	0.212	0.424
		Global *P* = 0.680		Global *P* = 0.680		Global *P* = 0.601		Global *P* = 0.345	

^a^Haplotype frequencies were inferred using the EM algorithm within the haplo.stats R package; haplotypes are not listed if all the estimated frequencies are <0.02 in controls, patients with stroke

^b^ *P* value based on haplotype-specific score tests

^c^Constituted by SNPs rs2200733 and rs10033464

## Meta-analysis

### Data extraction process

**Fig. 1:**
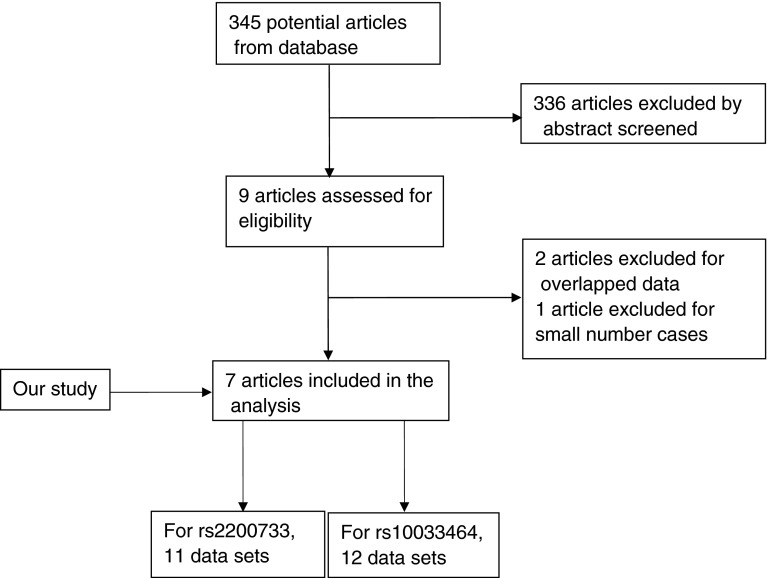
Flow chart of the selection of articles included in this meta-analysis

We initially obtained 345 potential articles, among which most were excluded for no relevance to our analysis after screening abstract. Nine articles remained to assess the full-text. Three articles then were removed because overlapping data [10, 13] and small number cases [14]. Finally, seven articles (combining our study) including 11 data sets for rs2200733 and 12 data sets for 10033464 met the inclusion criteria (Fig. 1).

### Association of rs2200733 polymorphism with the risk of stroke

**Fig. 2:**
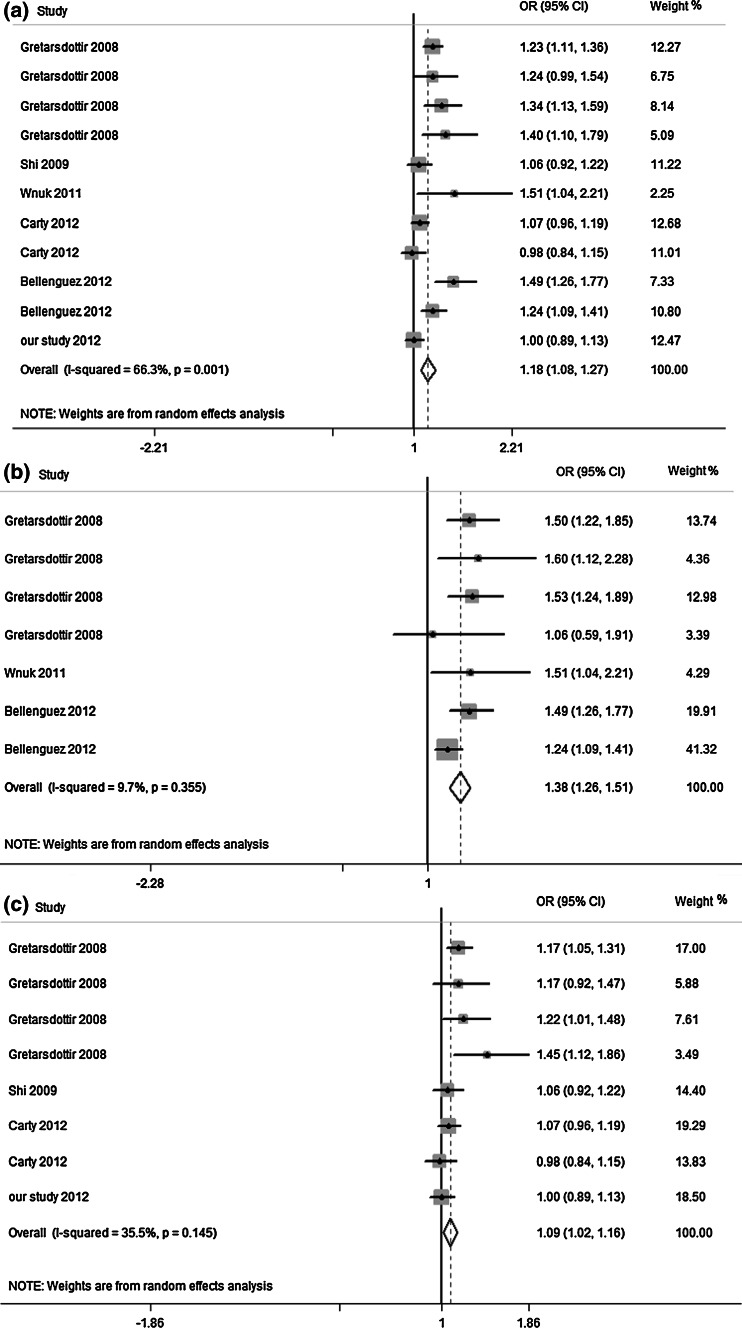
Meta-analysis of the association between rs2200733 and overall stroke, CE stroke and non-CE stroke. The squares and horizontal lines refer to the study-specific OR and 95 % CI. **a** Meta-analysis plot of association between rs2200733 and overall stroke. **b** Meta-analysis plot of association between rs2200733 and CE stroke. **c** Meta-analysis plot of association between rs2200733 and non-CE stroke

A total of 11 data sets from six studies (including our study) for rs2200733 polymorphism were involved in our meta-analysis containing 13,764 cases and 73,527 controls. The characteristics of these studies were shown in Table 4. In detail, seven data sets containing 3,825 cases and 40,980 controls described the association for CE stroke, as well as eight data sets (9,939 cases and 60,486 controls) for non-CE stroke. As shown in Fig. 2a, a random-effects model was performed for meta-analysis and generated a combined allelic OR of 1.18 for risk allele (95 % CI, 1.08–1.27) with heterogeneity (Q = 29.67, I^2^ = 66.3 %). When restricted to CE stroke subgroup studies, there was significant association between rs2200733 and the risk of CE stroke (OR 1.38, 95 % CI 1.26–1.51) (Fig. 2b) and no obvious heterogeneity was found (Q = 6.64, I^2^ = 9.7 %). Figure 2c showed the association between rs2200733 and non-CE stroke (OR 1.09, 95 % CI 1.02–1.16) was marginal and the heterogeneity of this meta-analysis reduced (Q = 10.86, I^2^ = 35.5 %).

**Table 4:** Studies included in this meta-analysis

Study, year	Ethnicity	No. of case/control	RS2200733			RS10033464		
			T frequency (case/control)	OR (95 % CI)	*P*	T frequency (case/control)	OR (95 % CI)	*P*
Gretarsdottir [7]	Iceland	1,943/25,708	0.142/0.119	1.23 (1.11–1.36)	5.4 × 10^−5^	0.085/0.082	1.07 (094–1.21)	0.3
	Sweden	1,060/724	0.119/0.098	1.24 (0.99–1.54)	0.06	0.111/0.114	1.00 (0.81–1.23)	0.99
	Germany-S	1,174/1,175				0.081/0.091	0.90 (0.73–1.11)	0.33
	Germany-W	1,391/1,107	0.146/0.114	1.34 (1.13–1.59)	0.00065	0.097/0.091	1.11 (0.92–1.35)	0.28
	UK	654/760	0.119/0.088	1.4 (1.10–1.79)	0.007	0.086/0.086	1.04 (0.80–1.36)	0.77
Shi [12]^a^	Chinese Han	811/688	0.496/0.511	1.06 (0.92–1.22)	0.43			
Lemmens [11]	Australia	588/496				0.086/0.093	0.92 (0.69–1.24)	0.6
	Austria	893/852				0.087/0.094	0.87 (0.69–1.11)	0.27
	Belgium	512/693				0.088/0.099	0.88 (0.66–1.16)	0.37
	Poland	1,116/570				0.086/0.081	1.06 (0.82–1.38)	0.64
	Spain	490/539				0.098/0.084	1.20 (0.87–1.65)	0.27
	Sweden	600/600				0.101/0.101	0.99 (0.76–1.30)	0.95
Wnuk [15]	Polish	301/428	0.204/0.155	1.51 (1.04–2.21)	0.03			
Carty [9]^b^	EA	3,239/23,279	/	1.07 (0.96–1.19)	0.201			
	AA	655/6,951	/	0.98 (0.84–1.15)	0.849			
Bellenguez [8]	European	790/5,972	/	1.49 (1.26–1.77)	3.64 × 10^−6^			
	European + American	1,532/6,281	/	1.24 (1.09–1.41)	3.99 × 10^−4^			
Our study 2012	Chinese Han	1,388/1,629	0.462/0.473	1.00 (0.89–1.13)	0.982	0.212/0.209	1.06 (0.92–1.22)	0.445

Germany-S, recruited from Department of Neurology, Klinikum Grosshadern, University of Munich, Munich, Germany; Germany-W, recruited from Westphalia region, Germany

*UK* United Kingdom, *EA* European Americans, *AA* African-Americans

^a^Ischemic stroke patients impossible to classify into other sub-categories

^b^Stroke patients without subgroup information and from following up study and impossible to classify into other sub-categories

### Association of rs10033464 and the risk of stroke

**Fig. 3:**
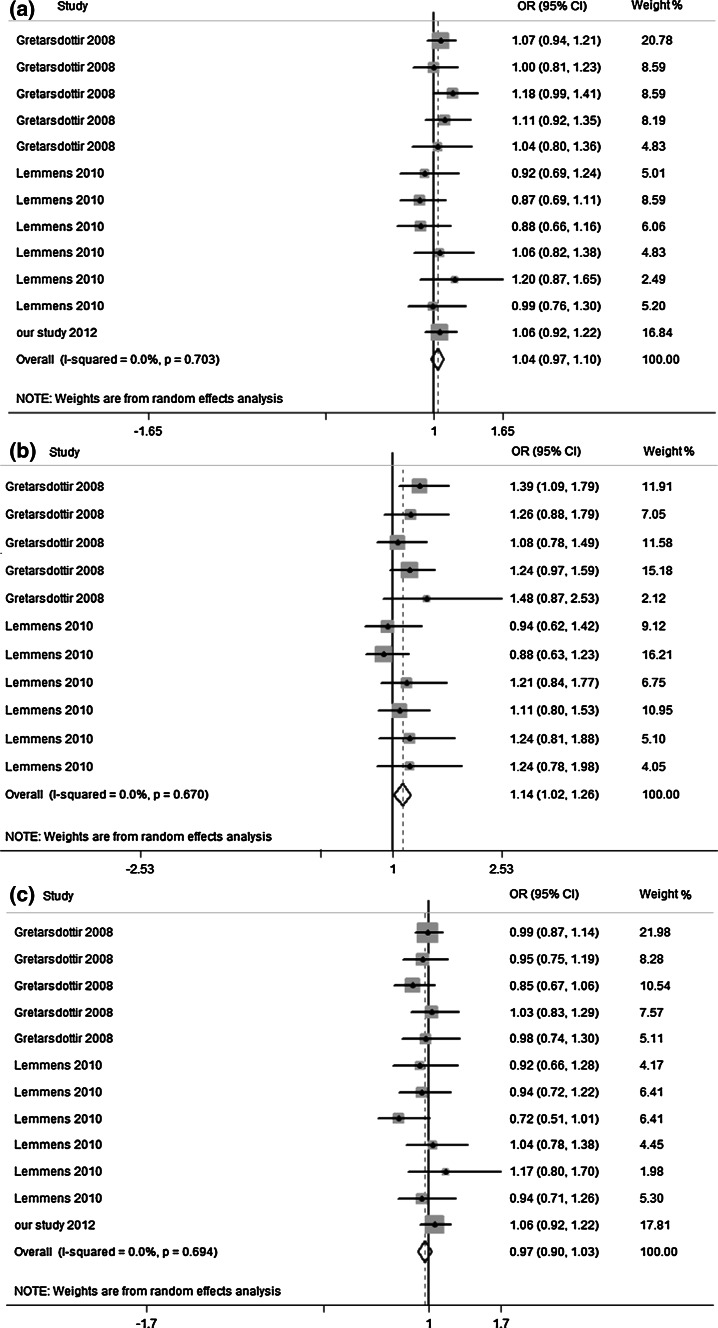
Meta-analysis of the association between rs10033464 and overall stroke, CE stroke and non-CE stroke. The *squares* and *horizontal lines* refer to the study-specific OR and 95 % CI. **a** Meta-analysis plot of association between rs10033464 and overall stroke. **b** Meta-analysis plot of association between rs10033464 and CE stroke. **c** Meta-analysis plot of association between rs10033464 and non-CE stroke

For rs10033464, 12 data sets (including our study) were available including 11,809 stroke patients and 34,853 control subjects: 11 of them had subgroups about stroke (CE stroke and non-CE stroke) and one cohort just discussed the relation to non-CE stroke (supplementary Table 2). Figure 3 respectively presented the association among rs10033464 and overall stroke, CE stroke, non-CE stroke. Obviously, Fig. 3a showed no association was found between rs10033464 and overall stroke (OR 1.04, 95 % CI 0.97–1.10) without heterogeneity (Q = 7.13, I^2^ = 0.0 %). However, subgroup analysis demonstrated that rs10033464 was significantly associated with CE stroke (OR 1.14, 95 % CI 1.02–1.26) without heterogeneity (Q = 7.58, I^2^ = 0.0 %) (Fig. 3b). No association was found for non-CE stroke (OR 0.97, 95 % CI 0.90–1.03) (Fig. 3c).

### Publication bias and sensitivity analysis

We further investigated the publication bias of every meta-analysis about rs2200733 and rs10033464 using Begg test and funnel plot (supplementary Fig. 1a–f). There was no evidence of publication bias (all *P* > 0.05). The sensitivity analysis also illuminated that none of including studies or cohorts influenced the stability of pooled ORs (supplementary Fig. 2a–f).

## Discussion

The two SNPs (rs2200733, rs10033464) on chromosome 4q25 were first found to be strongly associated with atrial fibrillation (AF) in European descent by Gudbjartsson et al. [19]. Another GWAS study verified these two variants also contributed to ischemic stoke, especially cardioembolic stroke [7]. However, the mechanism whereby the genetic variants exert their same effects on these two related phenotypes remains to be elucidated. The most possible explanation might be that the 4q25 locus associates with AF and therefore indirectly also associates with CE stroke.

We carried out a case–control study and failed to replicate the relevance of two SNPs and stroke in Chinese Han population. This discrepancy may be explained by the following reasons: (1) cardioembolic stroke as well as stroke patients with AF were excluded from our study; (2) these strongest signals may reflect genuine susceptibility effects on cardioembolic stroke but not other subtypes; (3) due to small sample size, we may lack enough power to detect the associations between these two SNPs and stroke. (4) genetic heterogeneity in different population. In China, among all subtypes of stroke, intracerebral hemorrhage accounts for 20–40 % of strokes in the Chinese population. In contrast, the majority (80–90 %) of strokes in most western populations are cerebral infarctions [20]. The reasons for the high incidence of stroke, especially the hemorrhagic subtype, among the Chinese population, indicating different genetic background are more implicated in the pathogenesis of disease development. Furthermore, the distributions of these two SNPs were different between various ethnic populations (for example, the frequency of T allele for rs2200733 in Chinese population is 0.537 compared to 0.104 in European cohorts based on Hapmap database) and there was population-specific genetic effect as a result of gene–gene and gene-environment interactions.

Since the first description of the association between 4q25 and stroke, a number of replication studies with conflicting results appeared. To date, there still was not final and coherent conclusion. Hence, combining our own cohort, we performed a meta-analysis for SNPs rs2200733 and rs10033464, respectively. Our meta-analysis harbored impressively large sample size from different studies, so the results seemed to be more reliable and stable than those from single study. However, there are limitations in it: (1) All included literatures were published in English or Chinese, therefore, we might lose some articles meeting our inclusion criteria but issued in other languages. (2) We only recruited relevant papers which had been published, so it was possible to miss some not published and inevitably to cause publication bias. (3) The studies included in our meta-analysis were all case–control studies or population-based studies, which precluded the further comments on cause–effect relationship. (4) Although we respectively carried out meta-analysis in CE stroke subgroup and non-CE stroke subgroup, not all included associated literatures had the data about subgroups. To some extent, the meta-analysis results of non-CE stroke subgroup were likely influenced by phenotypic misclassification. In view of this, it seemed plausible that our result that rs2200733 marginally associated with non-CE stroke susceptibility was not exactly true. The relevance between rs2200733 and overall stroke was also affected by CE stroke. So, we seem to conclude that rs2200733 only led to CE stroke. (5) The population in our meta-analysis most was from Europe and we failed to find any evidence to report the association between these two SNPs and cardioembolic stroke in Chinese Han population after extensively searching the literatures, thus studies undergoing in other countries or places (especially in China) should be needed. (6) due to no association between these two SNPs and AF were determinate in present study, we did not know whether the association remained after excluding the CE stroke patients with AF.

## Conclusion

In conclusion, by combing all available data from genetic studies on 4q25 and stroke, we confirmed rs2200733 and rs10033464 both were associated with CE stroke. However, the molecular basis under it still needs to be elucidated and well-designed studies with a large number of subjects from different countries and regions should be conducted in the future.

## Electronic supplementary material

Below is the link to the electronic supplementary material.

Supplementary material 1 (DOC 284 kb)
