# Supplementary material for: Rs2200733 and rs10033464 on chromosome 4q25 confer risk of cardioembolic stroke: an updated meta-analysis
Source: Mol Biol Rep. 2013 Sep 25;40(10):5977–85. doi: 10.1007/s11033-013-2707-z (PMC3824842; doi:10.1007/s11033-013-2707-z)
Supplement: Supplementary file 1 — Supplementary material 1 (DOC 284 kb) [file 11033_2013_2707_MOESM1_ESM.doc]

**Supplementary material**

**Rs2200733 and rs10033464 on chromosome 4q25 confer risk of cardioembolic stroke: an updated meta-analysis**

Yanyan Cao ^a^,*, Fei Ma^a^,*, Yan Wang^a^, Dao Wen Wang^b^, Hu Ding^a,b^, ,

^a^Institute of Hypertension and Department of Internal Medicine, Tongji Hospital, Tongji Medical College, Huazhong University of Science and Technology;

^b^Genetic diagnosis Center, Tongji Hospital, Tongji Medical College, Huazhong University of Science and Technology, Wuhan 430030.

*Yanyan Cao, Fei Ma contributed equally to this work.

Corresponding Author:

Hu Ding, MD, PhD or Dao Wen Wang , MD, PhD

Institute of Hypertension and Department of Internal Medicine, Tongji Hospital, Tongji Medical College, Huazhong University of Science and Technology 1095# Jiefang Ave, Wuhan 430030 People’s Rep. of China

Phone & Fax: 86-27-8366-2827

Email: [huding@tjh.tjmu.edu.cn](mailto:huding@tjh.tjmu.edu.cn) or [dwwang@tjh.tjmu.edu.cn](mailto:dwwang@tjh.tjmu.edu.cn)

**Legends for figures**

**Supplementary Figure 1. Funnel plot of the meta-analysis for the relationship between 4q25 and stroke.** logOR, natural logarithm of the OR; s.e. of logOR, standard error of logOR. **A**: Begg’s funnel plot for the meta-analysis of the association between rs2200733 and overall stroke. **B**: Begg’s funnel plot for the meta-analysis of the association between rs2200733 and CE stroke. **C**: Begg’s funnel plot for the meta-analysis of the association between rs2200733 and non-CE stroke. **D**: Begg’s funnel plot for the meta-analysis of the association between rs10033464 and overall stroke. **E**: Begg’s funnel plot for the meta-analysis of the association between rs10033464 and CE stroke. **F**: Begg’s funnel plot for the meta-analysis of the association between rs10033464 and non-CE stroke.

**Supplementary Figure 2. Sensitivity analyses of the two SNPs (rs2200733 and rs10033464) by omitting one cohort or one study at one time and calculating the pooled ORs of remaining cohorts or studies.** **A**: Sensitivity analysis of rs2200733 and overall stroke. **B**: Sensitivity analysis of rs2200733 and CE stroke. **C**: Sensitivity analysis of rs2200733 and non-CE stroke. **D**: Sensitivity analysis of rs10033464 and overall stroke. **E**: Sensitivity analysis of rs10033464 and CE stroke. **F**: Sensitivity analysis of rs2200733 and non-CE stroke.

| **Supplementary Table 1. TaqMan primer and probe sequences** | | | |
| --- | --- | --- | --- |
| **SNPs** | **Primer (5’→3’)** | **Allele** | **Allelic Probes^b^** |
| rs2200733 | Forward AGTAATTCTGCCTTGGTGGTACTTG | C | FAM-TTTTGATCAGAGAAAAT-MGB |
|  | Reverse CGGTTAGAATCTCACACTGTGAATG | T | HEX-ATTTTGATTAGAGAAAATTA-MGB |
| rs10033464^a^ | Forward TGAAGGTTATCCTCTTCCCTTTACTC | G | FAM-AAGCACTTCTTTCTTGACT-MGB |
|  | Reverse CAACTTTAAAGAAGGTTTGATTGTGTCT | T | HEX-AAGCACTTATTTCTTGACTC-MGB |
| ^a^Probes are designed to the reverse strand;  ^b^FAM, 6-carboxyfluorescein; HEX, hexachloro-6-carboxyfluorescein; MGB, minor groove binder probe. | | | |

| **Supplementary Table2. Studies including CE stroke or non-CE stroke selected into our meta-analysis** | | | | | | | | | | | |
| --- | --- | --- | --- | --- | --- | --- | --- | --- | --- | --- | --- |
| **SNPs** | **Study, year** | **Ethnicity** | **CE stroke** | | | | **non-CE stroke** | | | |  |
|  |  |  | **No. of Case/Control** | **OR(95%CI)** | **T frequency (case/control)** | ***P*** | **No. of Case/Control** | **OR(95%CI)** | **T frequency (cases/controls)** | ***P*** |  |
| rs2200733 | Gretarsdottir, 2008. | Iceland | 385/25708 | 1.50(1.22-1.85) | 0.164/0.119 | 0.00011 | 1558/25708 | 1.17(1.05-1.31) | 0.136/0.119 | 0.0062 |  |
|  |  | Sweden | 185/724 | 1.60(1.12-2.28) | 0.144/0.098 | 0.011 | 875/724 | 1.17(0.92-1.47) | 0.114/0.098 | 0.19 |  |
|  |  | Germany-W | 554/1107 | 1.53(1.24-1.89) | 0.161/0.114 | 8.25×10^-5^ | 837/1107 | 1.22(1.01-1.48) | 0.136/0.114 | 0.043 |  |
|  |  | UK | 78/760 | 1.06(0.59-1.91) | 0.090/0.088 | 0.84 | 576/760 | 1.45(1.12-1.86) | 0.123/0.088 | 0.004 |  |
|  | Shi, 2009^a^ | Chinese Han |  |  |  |  | 811/688 | 1.06(0.92-1.22) | 0.496/0.511 | 0.43 |  |
|  | Wnuk, 2011 | Polish | 301/428 | 1.51(1.04-2.21) | 0.204/0.155 | 0.03 |  |  |  |  |  |
|  | Carty, 2012^b^ | EA |  |  |  |  | 3239/23279 | 1.07(0.96-1.19) | / | 0.201 |  |
|  |  | AA |  |  |  |  | 655/6951 | 0.98(0.84-1.15) | / | 0.849 |  |
|  | Bellenguez, 2012 | European | 790/5972 | 1.49(1.26-1.77) | / | 3.64×10^-6^ |  |  |  |  |  |
|  |  | European+American | 1532/6281 | 1.24(1.09-1.41) | / | 3.99×10^-4^ |  |  |  |  |  |
|  | our study, 2012 | chinese |  |  |  |  | 1388/1629 | 1.00(0.89-1.13) | 0.462/0.473 | 0.982 |  |
| rs10033464 | Gretarsdottir, 2008 | Iceland | 385/25708 | 1.39(1.09-1.79) | 0.105/0.082 | 0.0088 | 1558/25708 | 0.99(0.87-1.14) | 0.08/0.082 | 0.91 |  |
|  |  | Sweden | 185/724 | 1.26(0.88-1.79) | 0.131/0.114 | 0.201 | 875/724 | 0.95(0.75-1.19) | 0.107/0.114 | 0.64 |  |
|  |  | Germany-S | 296/1175 | 1.08(0.78-1.49) | 0.092/0.091 | 0.66 | 878/1175 | 0.85(0.67-1.06) | 0.078/0.091 | 0.15 |  |
|  |  | Germany-W | 554/1107 | 1.24(0.97-1.59) | 0.105/0.091 | 0.086 | 837/1107 | 1.03(0.83-1.29) | 0.092/0.091 | 0.78 |  |
|  |  | UK | 78/760 | 1.48(0.87-2.53) | 0.122/0.086 | 0.15 | 576/760 | 0.98(0.74-1.3) | 0.081/0.086 | 0.89 |  |
|  | Lemmens, 2010 | Australia | 196/496 | 0.94(0.62-1.42) | 0.087/0.093 | 0.75 | 392/496 | 0.92(0.66-1.28) | 0.085/0.093 | 0.61 |  |
|  |  | Austria | 304/852 | 0.88(0.63-1.23) | 0.084/0.094 | 0.46 | 589/852 | 0.94(0.72-1.22) | 0.089/0.094 | 0.64 |  |
|  |  | Belgium | 172/693 | 1.21(0.84-1.77) | 0.118/0.099 | 0.31 | 340/693 | 0.72(0.51-1.01) | 0.073/0.099 | 0.057 |  |
|  |  | Poland | 411/570 | 1.11(0.80-1.53) | 0.089/0.081 | 0.53 | 705/570 | 1.04(0.78-1.38) | 0.084/0.081 | 0.81 |  |
|  |  | Spain | 195/539 | 1.24(0.81-1.88) | 0.101/0.084 | 0.32 | 295/539 | 1.17(0.80-1.70) | 0.096/0.084 | 0.41 |  |
|  |  | Sweden | 98/600 | 1.24(0.78-1.98) | 0.122/0.101 | 0.37 | 502/600 | 0.94(0.71-1.26) | 0.096/0.101 | 0.69 |  |
|  | our study, 2012 | Chinese Han |  |  |  |  | 1388/1629 | 1.06(0.92-1.22) | 0.212/0.209 | 0.445 |  |
| Germany-S, recruited from Department of Neurology, Klinikum Grosshadern, University of Munich, Munich, Germany; Germany-W, recruited from Westphalia region, Germany; UK, United Kingdom; EA, European Americans; AA, African-Americans; ^a^ischemic stroke patients impossible to classify into other sub-categories; ^b^stroke patients without subgroup information and from following up study and impossible to classify into other sub-categories. | | | | | | | | | | | |

**Supplementary Figure 1.** Funnel plot of the meta-analysis for the relationship between 4q25 and stroke.

**
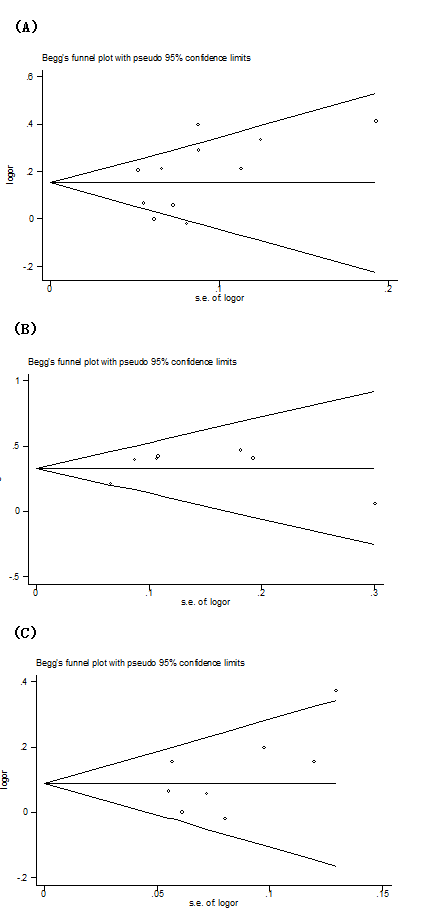
**

**
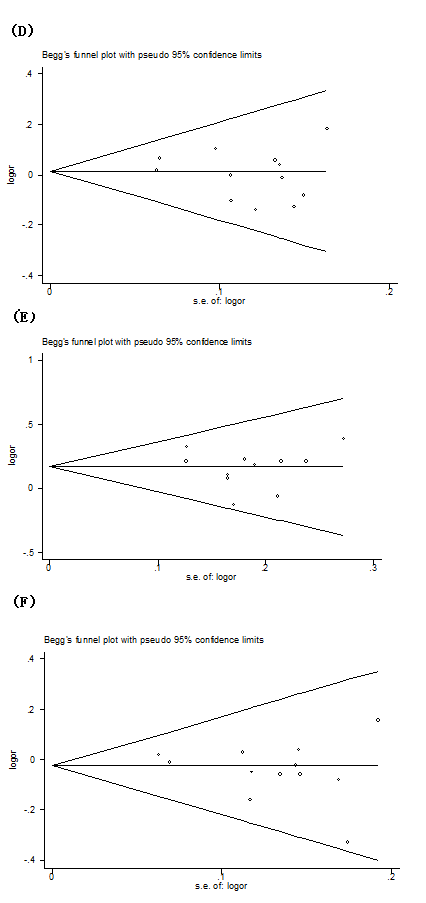
**

**Supplementary Figure 2.** Sensitivity analyses of the two SNPs (rs2200733 and rs10033464) by omitting one cohort or one study at one time and calculating the pooled ORs of remaining cohorts or studies.

**
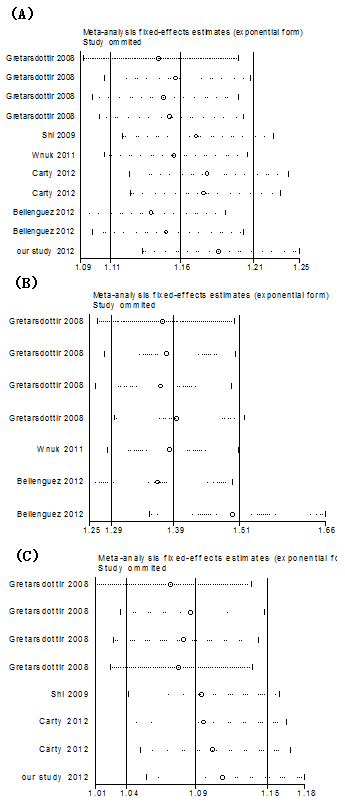
**

**
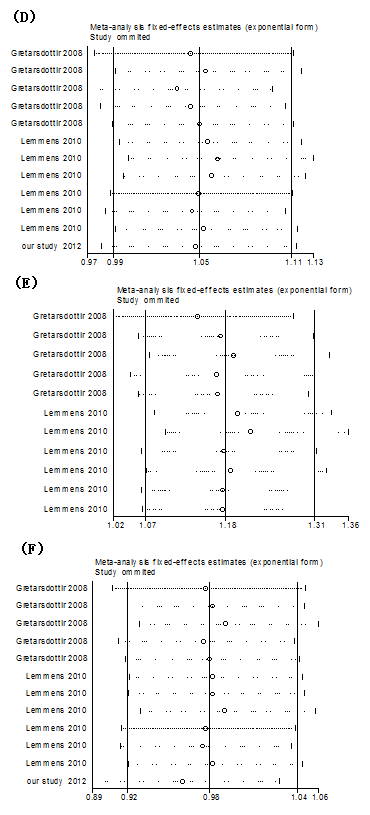
**
